# Antibiotic-Resistant* Acinetobacter baumannii* Increasing Success Remains a Challenge as a Nosocomial Pathogen

**DOI:** 10.1155/2016/7318075

**Published:** 2016-02-04

**Authors:** Ana Maria Gonzalez-Villoria, Veronica Valverde-Garduno

**Affiliations:** ^1^Escuela de Salud Pública de México, Instituto Nacional de Salud Pública, Avenida Universidad 655, 62100 Cuernavaca, MOR, Mexico; ^2^Departamento de Infección e Inmunidad, Centro de Investigaciones sobre Enfermedades Infecciosas, Instituto Nacional de Salud Pública, Avenida Universidad 655, 62100 Cuernavaca, MOR, Mexico

## Abstract

Antibiotic-resistant infectious bacteria currently imply a high risk and therefore constitute a strong challenge when treating patients in hospital settings. Characterization of these species and of particular strains is a priority for the establishment of diagnostic tests and preventive procedures. The relevance of* Acinetobacter baumannii* as a problematic microorganism in inpatient facilities, particularly intensive care units, has increased over time. This review aims to draw attention to (i) the historical emergence of carbapenem-resistant* Acinetobacter baumannii*, (ii) the current status of surveillance needs in Latin America, and (iii) recent data suggesting that* A. baumannii* continues to spread and evolve in hospital settings. First, we present synopsis of the series of events leading to the discovery and precise identification of this microorganism in hospital settings. Then key events in the acquisition of antibiotic-resistant genes by this microorganism are summarized, highlighting the race between new antibiotic generation and emergence of* A. baumannii* resistant strains. Here we review the historical development of this species as an infectious threat, the current state of its distribution, and antibiotic resistance characteristics, and we discuss future prospects for its control.

## 1. Introduction

The first report of* Acinetobacter* infections in a hospital setting, within an intensive care unit (ICU), dates back to the 1960s [1]. Phenotypic tests were initially used to ascribe gender to the organism causing these infections. Identification at the species level was only possible when molecular tests were employed. Difficulties in species identification may have caused delays in recognizing the microorganism and hence the impact of* Acinetobacter baumannii* as a nosocomial pathogen was underestimated. The persistent presence of* A. baumannii* in the hospital sector allowed it to come into contact with antibiotics. These environmental conditions exerted such a selective pressure that successful clones with particular antibiotic resistance characteristics emerged. Previous reviews have focused on* A. baumannii* as a successful pathogen, and its biological aspects, epidemiology, pathogenicity factors, and global spread have also been addressed. Here we review the increase of antibiotic-resistant* Acinetobacter* in recent reports and summarize the multilateral work being carried out in order to control this pathogen. As multinational networks have emerged based on the information provided by these reports, we update the available information and give an example of the importance of local reports. This review is organized as a timeline divided in three periods. The first period includes the decades of the 60s and 70s when the* Acinetobacter* gender started to be reported in hospitals. The second period includes the decades of the 80s and 90s during which* baumannii* species was determined and the reports of the organism's carbapenem resistance started. The last period includes years 2000 to 2015, during which World Health Organization (WHO) has issued calls for containment of antimicrobial resistance.

## 2. History of* A. baumannii* Species

The genus* Acinetobacter* was first described in 1911. The bacterium isolated from soil was called* Micrococcus calcoaceticus*,* Achromobacter*,* Alcaligenes*,* Bacterium anitratum*,* Moraxella glucidolytica*,* Neisseria winogradsky*,* Alcaligenes haemolysans*,* Mima polymorpha*, and* Moraxella lwoffii* [2]. The current designation of the genus* Acinetobacter* (from the Greek* akinetos*, meaning nonmotile) was proposed in 1954 by Brisou and Prevot and it was accepted in 1968 [3]. Finally, in 1974, this designation was included in* Bergey's Manual of Systematic Bacteriology*, where it was described as having only one species:* Acinetobacter calcoaceticus* [4]. Later, in 1986, Bouvet and Grimont observed inconsistencies in the application of phenotypic tests to determine the genus and species of* Acinetobacter*. These inconsistencies arose due to the fact that the members of this genus have different catabolic pathways that allow them to adapt to most substrates [5]. DNA hybridization studies were then introduced to determine the different species, and the homology groups with more than 70% DNA-DNA relatedness were called genomic species. Currently, there are 32 genospecies known. The complex* A. calcoaceticus*-*baumannii* includes four genospecies: genospecies 1,* A. calcoaceticus*; genospecies 2,* A. baumannii*; genospecies 3,* A. pittii*; and genospecies 13TU,* A. nosocomialis*. Among these,* A. baumannii* is the most important in the clinical context [6, 7], since it is the most frequently isolated in nosocomial infections and also the one associated with the highest mortality rate [8, 9].

## 3. Increasing Impact of* A. baumannii* at a Global Scale

It is difficult to determine the presence of* A. baumannii* in nosocomial infections in reports before the year 1974. At that time, there was a specific designation for a single species:* A. calcoaceticus*. Moreover, the routine laboratory diagnostic identification was carried out by phenotypic methods that are not useful for species designation. Particularly, these methods do not allow a distinction within the* calcoaceticus-baumannii* complex. Here we use identification and antibiotic resistance data arising from hospital settings where the pathogen has become successful.

**Figure 1:**
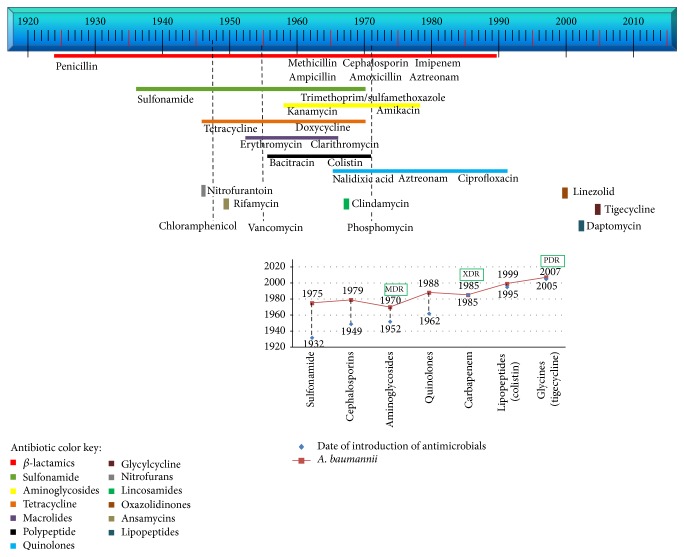
Top diagram shows dates of introduction of antimicrobials; relevant examples are indicated below colored lines for some groups. Dashed lines indicate antibiotics without group. Insert graph shows the date of introduction of antimicrobials, date of first reports of antimicrobial resistance in* A. baumannii*, and the emergence of multidrug-resistant (MDR), extensively drug-resistant (XDR), and pandrug-resistant (PDR) bacterial strains. Dashed lines indicate interval between antibiotic introduction and first report of resistance in* A. baumannii*.

A key question to understand the origin and impact of* A. baumannii* in hospital settings is the following: How did it become a pathogen? Olson has pointed out at least three contributing elements. First, water is an important reservoir for clinically significant Gram-Negative Nonfermenting (GN-NF) bacteria. Second, this species is a water-related organism, and solutions as well as surgical, medical, and clinical instruments can harbor high levels of bacteria. With bacteria already present, debilitated patients became susceptible to infection by this opportunistic microorganism, giving rise to nosocomial outbreaks [10]. Early* Acinetobacter* outbreaks were easily controlled with common antibiotics including *β*-lactams and sulfonamides. However, these treatments became ineffective very soon, coincident with early reports of emerging microbial resistance to these antibiotics. Unfortunately, since the 1970s, microbial resistance has only increased; at the same time, the creation of effective new antibiotics has declined (Figure 1). Emergence of such antibiotic-resistant strains has made the management of patients and the containment and control of outbreaks more difficult. These difficulties also increase morbidity, mortality, and hospital costs, making these outbreaks a challenge for public health systems [11]. The emergence of* A. baumannii* as a threat in hospital settings has common historical developments in diverse geographical locations worldwide. One of the most relevant events, besides the refinement of laboratory techniques to identify this species, is the acquisition of antibiotic resistance.

## 4. Emergence and Establishment of* A. baumannii* as a Nosocomial Microorganism: 1960s and 1970s

At this period in time,* A. baumannii* emerged as a microorganism causative of nosocomial infections that were easily managed. Successful treatment of those nosocomial infections was achieved simply with *β*-lactams. These reports arose mainly from hospitals located in Europe and the United States. The earliest reports of infections caused by this organism in a hospital setting came from Europe. Computer analysis of bacteriological data collected in 1965 in a German hospital revealed the presence of the* Acinetobacter* genera. In this case,* A. anitratus* was found to be present in blood culture samples, and no antimicrobial resistance was reported. By the end of the 1970s, some other countries reported the pathogen as a cause of infection outbreaks, also detected in blood culture samples [1, 12]. *β*-lactams and sulfonamides were used as an effective treatment for these infections up to 1975. That year,* A. calcoaceticus* was found to be the cause of an outbreak, despite the fact that it has been considered a low pathogenic microorganism. The microorganism causing this hospital epidemic showed a noticeable reduction in susceptibility to those antibiotics, thus explaining its higher impact [13].

Both in the United States and in Europe, earlier reports also date back to 1965. That year, a review on the* Moraxella* group included a report where* Acinetobacter* was described as belonging to the Gram-negative bacilli causing pneumonia [14, 15]. Latin American countries did not take long to begin reporting findings of this organism as a cause of nosocomial infections, starting in 1979 [7, 16]. Unfortunately, by the end of the 1970s,* Acinetobacter* was reported to be resistant to sulfonamides, *β*-lactams, and aminoglycosides. These antibiotics were previously considered the treatment of choice, but by this time they were no longer effective [17, 18]. Thus, in just about two decades,* Acinetobacter* became consolidated as a pathogen causing hospital outbreaks difficult to treat and contain on both sides of the Atlantic.

## 5. Outbreak Increase and Cumulative Antibiotic Resistance: 1980s and 1990s

This period was characterized by the specific designation of the genetic species within the complex and by the critical identification of* A. baumannii* species. In fact, it was only until 1986 that it became possible to attribute* Acinetobacter* infections specifically to* A. baumannii*. At the same time, outbreak analysis studies revealed increasing antimicrobial resistance (Figure 1).

The cumulative nature of antimicrobial resistance posed a new problem for the control and containment of these outbreaks. By the end of the 1990s, Asia and Latin America had integrated reports of outbreaks caused by antibiotic-resistant* A. baumannii* strains to their data. At this time, the geographical spread of outbreaks led to a change in the surveillance and in the levels of reporting. It became relevant to maintain both a regional surveillance and a worldwide surveillance of the outbreaks. This was carried out with a particular focus on the characterization of antibiotic resistance patterns based on molecular techniques.

In order to find ways to contain these outbreaks, European countries started the search for risk factors. These studies led to the establishment of an association between ventilator use and increased mortality with strains that were resistant to more than three antibiotic groups [19–21]. These strains were therefore designated as multidrug-resistant (MDR) microorganisms. At this point, carbapenems had emerged, and in 1985 they were used as the therapeutic option to treat infections by MDR bacteria. But unfortunately, despite the implementation of the imipenem treatment, resistance to these antibiotics was reported in this same year. Surprisingly, the enzymes responsible for such antibiotic resistance were the OXA-*β*-lactamases, already widespread among infectious microorganisms [22]. Those findings led to the implementation of association studies based on the phenotypic determination of the antibiotic resistance of outbreak isolates. A variety of methods were then applied to typify and compare hospital outbreak isolates as well as community isolates. These studies became necessary in order to generate new strategies to control the outbreaks [23–26]. The increasing number of cases associated with* Acinetobacter* in the United States made it necessary to establish a set of specific features associated with the* Acinetobacter* infections. This in turn led to the finding of an association with a diversity of risk factors, including ventilator use and previous antimicrobial therapy [27, 28]. An important observation was that the number of isolates was proportional to the increase in the number of antibiotics to which the microorganism was resistant. In general, the increased frequency of* A. baumannii* in clinical isolates correlated with a progressive increase in antibiotic resistance [21].

A key event in the characterization of* A. baumannii*'s impact as a microorganism causative of nosocomial infections was the species designation in 1986. This led to the realization that most infections attributed to* Acinetobacter* were in fact caused by* baumannii* species. It was then assumed that there was a single* Acinetobacter* species of clinical interest, and several biotypes were reported. However, the phenotypic methods used for species typing were producing inconsistent results. Therefore, molecular methods, such as plasmid characterization, were introduced in order to analyze the antibiotic resistance mechanisms. By the end of the 1980s, nosocomial reports characterizing* A. baumannii* infections had notably increased, and special focus on antibiotic resistance features uncovered then reduced susceptibility to imipenem [29–31]. At this period in time, there was also an increase in the reports of* Acinetobacter*-positive nosocomial isolates in Latin America. Despite the limitations to the identification, the first half of the 1990s produced the highest number of reports, although the pathogen was still being described as* A. calcoaceticus* variety* anitratu*s. These findings led to the introduction of better hygiene practices including thorough hand washing to prevent transmission within hospital settings [32–35]. It was only after 1990 that Asia started to produce reports of* A. baumannii*'s role in nosocomial infection outbreaks. Before 1990, reports from Asia were related only to environmental food-borne* Acinetobacter* strains. The first* A. baumannii* strain from a hospital setting had low pathogenicity. However, by 1994, multiresistant strains were being detected in Asia, and they became a great public health challenge, just as it was happening in other continents [36, 37].

## 6. Creation of Surveillance Networks: 2000 to 2015

**Figure 2:**
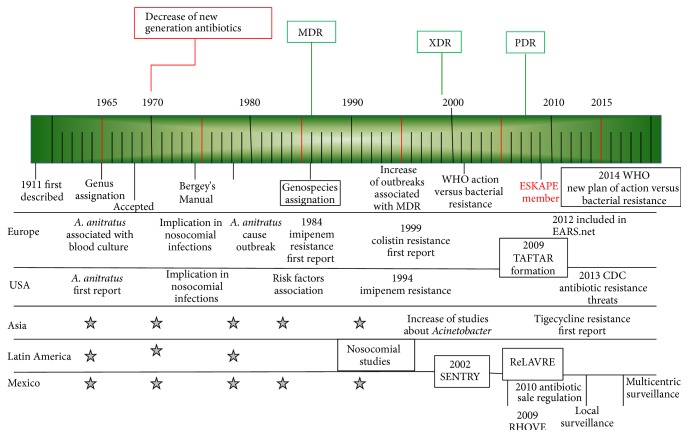
Brief history of the incorporation of* Acinetobacter baumannii* as one of the successful multidrug-resistant nosocomial pathogens. This has led to the strategic alliance of different countries and continents in monitoring resistant bacteria, and it has resulted in the formulation of a new action plan against bacterial resistance in 2015.

The most recent period has been characterized by the dissemination of carbapenem-resistant* A. baumannii* (CRAB) strains and the creation of international surveillance networks [6]. This has led to the implementation of measures to achieve the containment of outbreaks of this antibiotic-resistant pathogen, which has turned into a global priority. The first international call for containment was issued in 2001. WHO proposed a series of recommendations to slow down the emergence and reduce the spread of bacterial resistance; they were described in “Global Strategy for Containment of Antimicrobial Resistance,” published that year. This document considers that the surveillance of antimicrobial resistance should be carried out among common pathogens in the community as well as in hospital and other healthcare facilities [38]. It is then recommended that surveillance should be directed to microorganisms with high antibiotic resistance index. This group of microorganisms is designated by the acronym ESKAPE, and it includes* Acinetobacter*. The worldwide presence of antibiotic-resistant microbes has made surveillance shift from a local scale to a global one (Figure 2). Therefore, the challenge that emerges is to contain the dissemination of the resistant strains, leading to the formation of multinational surveillance networks. The first was the European Antimicrobial Resistance Surveillance System (EARSS) constituted in 1998 and renamed as European Antimicrobial Resistance Surveillance Network (EARS-Net) in 2010. This network is coordinated by the European Centre for Disease Prevention and Control (ECDC). The 2013 Annual Epidemiological Report showed a frequency of CRAB strains of 3.6% in healthcare-associated infections; 81.2% were carbapenem nonsusceptible [39].

In the United States, the national public health surveillance system is a collaborative project between CDC, FDA, USDA, and state and local health departments. The CDC together with the Interagency Task Force on Antimicrobial Resistance (ITFAR) coordinates different agencies for the containment of resistance. The 2013 CDC report shows that* Acinetobacter* was the cause of 2% of nosocomial infections that year. However, in critically ill patients on mechanical ventilators, it was responsible for about 7% of the infection cases. Moreover, 7,300 (63%) out of 12,000 annual* Acinetobacter* infections were MDR, which in turn has led to about 500 annual deaths attributed to these infections [40]. Acknowledging that the growing problem of bacterial resistance is a public health challenge, the USA (CDC) and the EU (ECDC) established the Transatlantic Taskforce on Antimicrobial Resistance (TAFTAR) in 2009. This network was created to foster international collaboration on the prevention and control of antimicrobial resistance (http://www.cdc.gov/drugresistance/tatfar/about.html) [41]. The highest levels of bacterial resistance to carbapenems were reported in Latin American countries. These findings resulted in the implementation of antimicrobial resistance surveillance at a national level in Brazil, Argentina, and Colombia, with special focus on uncovering molecular mechanisms [42, 43]. Additional studies provided by the SENTRY Antimicrobial Surveillance Program and other programs have reported different rates of resistance in Latin American countries. The highest resistance to imipenem was detected in Argentina (20%), followed by Colombia (14%) and Brazil (8.5%). The increase in MDR* A. baumannii* was noticed for the first time in 2001 by Gales and collaborators [44].

Despite a general increase of carbapenem-resistant* Acinetobacter* isolates, specific reports have rarely arisen from Chilean agencies. In Brazil, apparently decreasing rates were reported between 1997 and 2011 (13.6% to 2.2%) [45]. However, a study revealed a real increase in imipenem resistance: from 12.6% for the 1996-1997 period to 71.4% for the 2008–2010 period. Increases in Argentina and Chile were 84.9% and 50%, respectively [46]. In addition to the national surveillance systems, the Latin American Antimicrobial Resistance Surveillance Network (ReLAVRA-PAHO) was created. It specified that efforts should be made to carry out* Acinetobacter* surveillance in hospital settings [47]. This network includes 21 countries: Argentina, Bolivia, Brazil, Canada, Chile, Colombia, Costa Rica, Cuba, Ecuador, El Salvador, Guatemala, Honduras, Mexico, Nicaragua, Panama, Paraguay, Peru, Dominican Republican, USA, and Venezuela. Within this network, each country has its own system and not all the countries report the resistance profile of nosocomial bacteria.

Currently, some countries in Asia are carrying out surveillance at a national level. These include Malaysia, Thailand, Pakistan, India, and Taiwan, where* A. baumannii* is one of the most common pathogens [48]. In 2011, this microorganism was considered to belong to the group of pathogens characterized by carbapenem resistance [49]. The existence of the new carbapenemase NDM-1 was reported in 2008. This enzyme originated in India and was quickly disseminated among bacteria, including* A. baumannii* [50]. International studies have shown that Asia contains a large number of resistant Gram-negative bacteria (GNB) [51, 52]. However, most Asian countries are currently lacking antimicrobial-resistant bacteria surveillance systems [53].

The data collected by international networks have allowed researchers to generate a global picture of the circulating antibiotic-resistant strains. Recent studies have shown the presence of three successful clones called ribogroups or World Wide Types distributed in some countries: WWI, WWII, and WWIII [54]. International agreements to standardize methodologies and allow wide data comparison have led to the implementation of multilocus sequence typing (MLST). This analysis includes the characterization of seven housekeeping genes to define a sequence type (ST). The associations of these sequence types, where there is similarity among six to seven alleles, were defined as clonal complexes. For instance, it has been shown that clonal complex CC92, previously known as WWII, is the most distributed one around the world [55], followed by CC109 and CC87 (determined by the Oxford system). There are also international clones that can be found in a single continent: such is the case of CC636 (CC113) in Latin America [56].

The beginning of the 21st century was marked by a sharp increase in international mobility. The intense travelling across countries and continents has had a strong effect on the dissemination of infectious bacteria throughout the world [57]. This is particularly clear when the traveler is a sick person. For instance, Belgium received a sick patient travelling from Greece, and subsequently an outbreak caused by* A. baumannii* took place [58]. Another clear case is the occurrence of* A. baumannii* outbreaks after the arrival of military patients from Iraq [59]. However, patient transfer between hospitals is not the single cause of international dissemination. Medical tourism has also had a strong impact as a cause of antibiotic-resistant bacteria dissemination in Latin America and Europe [60].

## 7. Surveillance Networks: Advantages and Current Limitations

A positive outcome of the establishment of international surveillance networks is that member countries have to provide information. This baseline information is useful to understand the development and dissemination of antibiotic-resistant strains. Furthermore, the main positive impact perceived is the standardization of methods across countries and an improved laboratory capacity, both of which enhance the quality of testing. This is important because surveillance information allows the establishment of specific strategies targeting each country and/or region. These strategies can be designed according to impact, and they can be followed by particular indicators that can be measured at the surveillance centers belonging to the network [41]. Despite the progress achieved so far, bacterial resistance to antibiotics is still a major public health problem. Reports of carbapenem resistance continue to increase. Therefore, surveillance networks must act on a continuous basis and in a comprehensive way [41]. The sustainability of the surveillance networks depends on the support of the participant governments, including the on-going funding of network activities as well as adequate public health policies [61]. The current situation of bacterial resistance to antibiotics has alarmed the international medical community. In consequence, World Health Organization (WHO) has developed a new plan to address the challenge posed by antimicrobial resistance [61].

## 8. Current Challenges to Global Surveillance Integration

Different countries face different challenges; here we illustrate that although continuous surveillance programs are ideal, contributions of individual hospitals and research groups can provide valuable information. Here available data on Mexico is described as an example to illustrate the value of these efforts. An early limitation in the case of Mexico (1990s) was the use of phenotypic methods; as a consequence, such information should at least be used with caution when integrated with more recent data [13, 65]. A summary of more recent data regarding* A. baumannii* in this country is presented in Table 1. These data include surveillance carried out by individual hospitals; for instance, in a provincial hospital, MDR* A. baumannii* incidence was 74% for 2010 [64] and it became the second most frequently isolated pathogen [63]. Also, the mortality rate imputable to this organism was 14.5% [66]. Two additional studies were carried out by SENTRY: the 2003 study was inconclusive due to insufficient sampling, and the 2012 study showed that, at a national level, 16.7% of the isolates were imipenem-resistant [45, 46]. In this country, regional surveillance is usually reported by the Ministry of Health (*Secretaría de Salud* (SESA)) [67]. There are two additional programs, the epidemiological hospital network (RHOVE) [68] and the National Institute for Epidemiologic Reference (*Instituto Nacional de Referencia Epidemiológica* (INDRE)) program, as part of the Latin American network for monitoring resistance to antibiotics (ReLAVRE). However, no recent information on* A. baumannii* was available from these sources [67]. Nevertheless, the scarce data provided by a few studies reveal the presence of antibiotic-resistant* Acinetobacter* in hospital settings in Mexico. Furthermore, a multicenter study carried out recently provides valuable information to be integrated into the global picture [69]. This study reported the presence of carbapenem-resistant* A. baumannii* in various hospitals from different regions in this country. It found a predominance of the Iberian-American clonal complex CC636 and the international clonal complex CC92. It also reported a regional distribution of oxacilinases OXA-72 and OXA-239 in the North and South of Mexico, respectively. An important observation derived from this study is the presence of different STs constituting two clonal complexes among the hospitals included. It is important to note that this kind of distribution had been previously reported in the USA, in Brazil [70], and more recently in Argentina [71]. In addition, regional distribution of OXAs in different STs suggests a process of dissemination among hospitals in different regions. This distribution may have arisen as a consequence of increased patient mobility, highlighting the relevance for further efforts towards a more integral surveillance and application of preventive strategies. Data provided by this kind of studies can be integrated into the global picture, as they contribute valuable information for the international surveillance of antibiotic-resistant* A. baumannii* [69].

## 9. The Role of Surveillance and Reporting in Preventing the Consequences of Global Mobilization

Increased mobilization of people worldwide is an intrinsic component of globalization. The increase in medical tourism increases the probability of MDR bacteria exchange. A clear example is the case of Mexico, which is considered one of the top medical tourism destinations [72, 73]. As a consequence of frequent patient exchange, other countries have reported bacterial infections in patients arriving from Mexico [60]. Therefore, a key element in preventing global dissemination is the establishment of consistent surveillance programs capable of constant monitoring at sites with the highest patient exchange indices [57]. Since bacterial antibiotic resistance is a global challenge, it would be ideal to ensure the cooperation of international and national surveillance instances. Local data collection and prevention are relevant when possible, but they are insufficient to compose a picture of the national situation and its consequences at a global scale. Further efforts, including potentially compulsory surveillance and data gathering, are required in order to properly face this global challenge.

Bacteria causing specific diseases should be closely monitored through the establishment of precise programs. Such has been the case with tuberculosis: multidisciplinary action groups, like the Directly Observed Treatment Short-Course (DOTS) program, have established appropriate programs to prevent the spread of resistance. This strategy includes five important components: government, case detection, standardized treatment observed by healthcare workers, drug supply, and a standardized recording and reporting system. The implementation of a public health policy at an international level increases the potential success of the intervention [74]. The restricted sale of antibiotics is a unique public policy directed to stop the emergence of bacterial resistance in the community. The implementation of this policy is a key step in the global control of antibiotic resistance. However, additional factors may play important roles in limiting the positive effects of this policy. These potential factors may include the following: corruption or lack of law enforcement, governance, lack of surveillance, and lack of containment of the spread of resistant bacteria [75].

Multicenter collaboration among hospitals remains a relevant component of surveillance and its continuity should be supported. However, it is critical to start working towards the establishment of a national surveillance program as part of the Global Strategy for Containment of Antimicrobial Resistance in medical centers [38]. This global strategy has two critical aims. The first one is to prevent resistant bacteria dissemination; the second one is to prevent the accelerated emergence of new forms of resistance in order to avoid further restriction of therapeutic options. The main mechanism to contain nosocomial outbreaks has been the use of antibiotics. However, when antibiotics are rendered ineffective due to bacterial resistance, the possibility of an efficient containment becomes compromised. In order to propose, implement, and evaluate new specific containment interventions it is essential to gather specific data on MDR bacteria such as* A. baumannii* and make them available.

## Figures and Tables

**Table 1:** Surveillance studies carried out in Mexico.

Date (year of publication)	Type of surveillance	Region of study	Main results	Outbreak	Reference
2010	Local	Monterrey	Incidence of multidrug-resistant (MDR) *Acinetobacter*: 74%	−	[56]

2013	Local	Guadalajara	*Acinetobacter* endemicity and decreased susceptibility. Became one of the five microorganisms most frequently isolated in nosocomial infections.	+	[57]

2014	Local	Mexico City	Presence of ST758 and the new OXA-23 allele called OXA-239	+	[59]

2015	Local	Monterrey	Attributable mortality: 14.5%. Presence of new sequence type and OXA-58 and OXA-72	+	[60]

2015	Local	SLP	OXA-72 associated with plasmid.	+	[62]

2010	National	National^a^	No data accessible.	−	[63]

2011	National	National^a^	Frequency of 7% in nosocomial infections. No more data accessible.	−	[64]

2012	National	Guadalajara	Presence of OXA-24	−	[61]

2015	National	Multicentered	Predominance of clonal complexes CC636 and CC92. New OXA-469 allele found and regional distribution of OXA-72 and OXA-239 determined.	−	Submitted

^a^Included all the states in Mexico.

+, positive; −, negative; SLP, San Luis Potosí.
